# Interaction networks of macrophage glycolysis and inflammation in sepsis: mechanisms and therapeutic potential

**DOI:** 10.3389/fimmu.2026.1783409

**Published:** 2026-04-08

**Authors:** Peiyao Luo, Hao Liu, Ting Zhang, Wenfang He

**Affiliations:** 1 Department of Critical Care Medicine, The Second Xiangya Hospital, Central South University, Changsha, China; 2 Clinical Center for Gene Diagnosis and Therapy, The Second Xiangya Hospital of Central South University, Changsha, Hunan, China

**Keywords:** inflammation molecules, macrophages, metabolism, sepsis, the Warburg effect

## Abstract

Sepsis is a severe threat to human health with high mortality rates, but so far its pathogenesis is unclear and lacks effective therapeutic drugs. Macrophages function as one of the most important innate immune cells and play an integral role in the sepsis inflammatory process. Recently, studies have shown that its immune function is associated with the Warburg effect. The Warburg effect refers to the preferential metabolism of glucose to lactate by cells through aerobic glycolysis even with abundant oxygen. It has shown that increasing aerobic glycolysis promotes M1 polarization of macrophages to facilitate inflammation, whereas decreasing aerobic glycolysis can lead to M2 polarization and alleviated inflammation. Interestingly, it was demonstrated that not only does glycolysis affect inflammation, but inflammation in sepsis in turn affects glycolysis. Currently, there is no comprehensive review regarding this issue. Therefore, our review focuses on the mechanisms of the interaction between inflammation and macrophage glycolysis in sepsis. We will address both how inflammatory molecules affect the process of glycolysis in septic macrophages and how glycolytic enzymes and related metabolites contribute to inflammation. We also discuss the potential in targeting glycolysis for the treatment of sepsis. We hope to bring a new perspective to clinical practice.

## 1 Introduction

Sepsis is a life-threatening organ dysfunction caused by a dysregulated host response to infection (1). The incidence of sepsis in hospitalized patients can be as high as 4.1%, corresponding to an annual incidence rate of approximately 700 cases per 100,000 population. The mortality of sepsis patients is approximately one-fifth to three-tenths, compared to fifty percent for ICU sepsis patients combined with organ dysfunction (2–4). Although progress has been made in the diagnosis and treatment of sepsis in recent years, its high mortality rate is still a serious public health problem. Generally, the high mortality is directly related to severe organ dysfunction (5), which is mainly caused by excessive inflammation (6). In contrast, many anti-inflammatory drugs show little function in improving the prognosis of sepsis in both animal studies and clinical trials (7–9). Moreover, recent studies have confirmed that sepsis is characterized by sustained excessive inflammation and immune suppression (10), making the pathological process of sepsis more complicated. Therefore, it is important to explore the mechanism of immune dysfunction for the treatment of sepsis.

Macrophages are important components of the innate immune system and are distributed throughout several vital organs, which is related to sepsis-induced multi-organ dysfunction, such as sepsis-associated encephalopathy (SAE), acute lung injury (ALI), acute kidney injury (AKI), and sepsis-induced liver injury (11–14). Macrophages activate the immune system and defend the body against the invasion of pathogenic micro-organisms, while over-activated macrophages may disrupt the balance between inflammation and anti-inflammation, leading to tissue damage, and can even induce immunosuppression, exacerbating the pathological process of sepsis (15–17). This phenomenon is likely linked to macrophage polarization, as M1 macrophage polarization exacerbates inflammation in sepsis, whereas M2 polarization alleviates inflammation (18, 19). As the disease progresses from the inflammatory phase to the immunosuppressive phase, M1-type cells are gradually replaced by M2-type cells, indicating that the two types often coexist during the disease course (20). And there are other phenotypes that remain underexplored (21). Interestingly, recent studies have suggested that the Warburg effect may be involved in mediating macrophage polarization in sepsis (22, 23).

The Warburg effect refers to the metabolic process where cancer cells preferentially metabolize glucose to lactate via aerobic glycolysis, even though sufficient oxygen is available (24). Initially, studies of the Warburg effect focused on tumor metabolism and yielded substantial results. However, although the exact mechanism is unclear and deserves to be fully investigated, there is increasing evidence that the Warburg effect may also be involved in metabolic reprogramming to promote systemic inflammation and macrophage polarization in sepsis (25). The Enhanced Warburg effect is usually accompanied by macrophages toward M1 polarization, and reduced Warburg effect tends to shift them to M2 (26, 27). This process may be modulated by pro-inflammatory mediators (28) and mitochondrial dysfunction (29, 30). Contemporarily, a growing body of research has demonstrated that glycolysis, in turn, plays a significant role in mediating inflammatory responses during sepsis (31). However, to date, there has been no systematic review of the interaction mechanisms between inflammation and glycolysis of macrophages during the pathophysiological process of sepsis. Therefore, based on the critical function of macrophages in sepsis, our review elaborates on this topic in the hope of providing insights for future research in metabolic-targeted therapies in sepsis.

## 2 Inflammation molecules affecting glycolysis

Excessive inflammation frequently coincides with the activation of glycolysis (32, 33), and the specific molecular mechanisms underlying this process remain unknown. Studies have shown that several inflammatory molecules, such as nuclear factor kappa-B (NF-κB), hypoxia-inducible factor-1α (HIF-1α), and protein kinase B (AKT), are involved in the regulation of glycolysis in septic macrophages. Moreover, mitochondrial damage and cytokine release are also closely linked to the activation of glycolysis.

The interplay between these elements is complex and requires further investigation. In the following section, we will elaborate on this issue in detail.

### 2.1 HIF-1α

HIF-1α is a highly conserved member of the PER-ARNT-SIM (PAS) subfamily. When HIF-1α binds to the promoters of target genes with hypoxia response elements (HREs), these genes are transcribed involved in hypoxia adaptation, metabolism, and cellular functions (34). HIF-1α has been demonstrated to be relevant to elevated expression of inflammatory factors (32, 33). Notably, the stabilized HIF-1α plays a pivotal role in driving the upregulation of glycolytic metabolic pathways (34). Subsequent oncology-focused investigations have confirmed that HIF-1α is able to trigger the expression of glycolytic enzymes—including Hexokinases 2 (HK2) and phosphofructokinase (PFK) —within tumor tissues (35, 36).

Drawing on these experimental observations, relevant research efforts have now set out to probe the function of HIF-1α in aerobic glycolysis during sepsis. It has been demonstrated that the overexpression of HIF-1α in septic macrophages increases the expression of methyltransferase-like 3 (METTL3), elevates m6A levels on PFKM, and upregulates PFKM protein expression, thereby enhancing glycolysis (37). Whether HIF-1α promotes glycolysis through additional glycolytic enzymes in sepsis needs to be further investigated.

**Figure 1:**
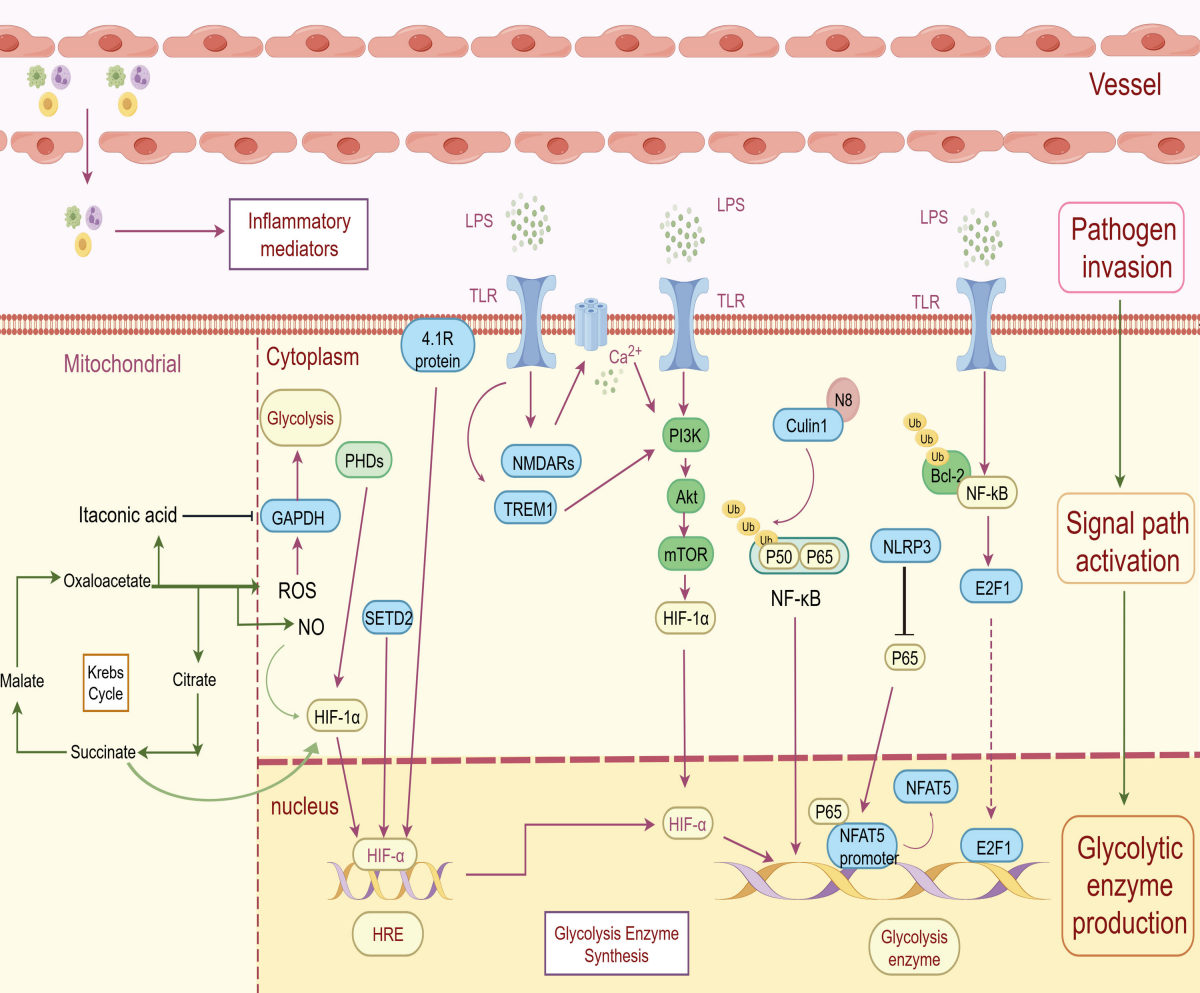
Inflammation-related molecules regulate the expression of glycolytic enzymes. HIF-1α promotes glycolysis enzyme expression as a transcription factor, while protein 4.1R and SETD2 work as the upstream molecules of HIF-1α to elevate the expression of glycolysis enzymes. Similarly, oxaloacetate promotes glycolysis by inducing NO production to increase HIF-1α activation, while succinate promotes glycolysis by inhibiting PHDs to stabilize HIF-1α. NMDARs increase Ca^2+^ accumulation, enhancing phosphorylation of PI3K at Y458 and AKT at T308. TREM-1 activates the PAM signaling pathway, promoting HIF-1α accumulation and nuclear translocation to increase glucose consumption and glycolysis. NF-κB could facilitate the upregulation of glycolysis through the transcription factor E2F1. NEDD8-mediated neddylation of Cullin1 could promote glycolysis and M1 polarization by enhancing the NF-κB p65 pathway. However, NLRP3 can inhibit the binding of nuclear NF-κB p65 to NFAT5 to reduce glycolysis gene expression, while Bcl-3 directly inhibits the activity of NF-κB. ROS, another production indirectly derived from citrate, promotes glycolysis through continuous oxidation of exposed methionine residues (M44) on GAPDH. Conversely, itaconic acid alkylates cysteine residue 22 on GAPDH, inhibiting its enzymatic activity and suppressing aerobic glycolysis. (by Figdraw).

In sepsis, HIF-1α is modulated by multiple upstream factors to affect its regulation of glycolysis. Protein 4.1R acts as an adaptor, linking membrane proteins to the cytoskeleton, and is involved in cell activation and cytokine secretion. It has been demonstrated to regulate HIF-1α-mediated glycolysis and promote M1 macrophage polarization in sepsis-induced liver injury (**Figure 1**) (38). Histone methyltransferase SETD2 is a methyltransferase catalyzing H3 lysine 36 trimethylation (H3K36me3). It regulates multiple biological processes by modulating H3K36 methylation. It can suppress HIF-1α expression through H3K36me3 catalysis in sepsis (39)(**Figure 1**) to suppress M1 macrophage polarization and glycolysis. Human umbilical cord mesenchymal stem cells (MSCs), by secreting extracellular vesicles (EVs), show beneficial effects in various inflammatory diseases. MSC-derived extracellular vesicles inhibit HIF-1α expression in LPS-stimulated primary Kupffer cells (40). Both result in significant suppression of glycolysis. These findings provide a theoretical basis for targeting HIF-1α in sepsis treatment.

Notably, the role of HIF-1α in glycolysis varies depending on cell states and macrophage subtypes (41). Under steady-state conditions, reduced HIF-1α expression lowers glycolytic activity in both alveolar and interstitial lung macrophages. This shows HIF-1α controls glycolysis in these two cell types. However, unlike interstitial lung macrophages, alveolar macrophages do not ramp up glycolysis in response to LPS under inflammatory conditions, no matter how much HIF-1α they produce. This points to distinct responses of different cell types to inflammatory microenvironments, though the exact mechanisms are still unknown.

### 2.2 PI3K-AKT-mTOR

The Phosphatidylinositol 3-kinases-protein kinase B-mammalian target of rapamycin (PI3K-AKT-mTOR or PAM) signaling pathway is a highly conserved, core signaling network in all higher eukaryotic cells (42). Many biological processes—such as cell survival, cycle progression, differentiation, senescence and metabolism—coordinate key events in inflammatory responses to damage and infection. All these processes are regulated by the activation state of Phosphatidylinositol 3-kinases (PI3Ks) (43). Like HIF-1α, recent research has confirmed that the PAM pathway is strongly activated during immune stimulation to regulate glycolysis. Luo Y’s team examined metabolism-related differentially expressed proteins in LPS-stimulated RAW2640.7 macrophages. Through phosphoproteomic analysis, they uncovered a new feature of acute inflammation—enhanced glycolysis and increased lipid synthesis. Meanwhile, KEGG pathway enrichment analysis emphasized the important function of the mammalian target of rapamycin (mTOR) signaling pathway (44).

This regulatory mechanism probably comes from the PAM pathway’s capacity to control the expression of key glycolytic enzymes in sepsis-induced macrophages. Deeply-researched glycolysis enzymes include HK2 (45), Pyruvate kinase M2 (PKM2) (46, 47), and lactate dehydrogenase A (LDHA) (48). The exact mechanism is still unclear, but recent studies suggest HIF-1α may act as a potential downstream mediator of the PAM pathway. N-methyl-d-aspartate receptors (NMDARs) are ionotropic glutamate receptors and are highly expressed in LPS-stimulated macrophages. This leads to Ca²^+^ buildup, promotes PAM pathway activation, and ultimately enhances glycolysis (**Figure 1**). When bone marrow-derived macrophages (BMDMs) are treated with NMDA—a major NMDAR agonist—PI3K phosphorylation and PKM2 phosphorylation increase significantly, along with a notable rise in HIF-1α protein levels (48). Triggering receptor expressed on myeloid cells-1 (TREM-1) is an activating immune receptor. It is constitutively expressed on monocytes/macrophages and neutrophils (49). It promotes HIF-1α accumulation and nuclear translocation through the PAM signaling pathway (**Figure 1**). This process increases glucose consumption and glycolysis in macrophages, while also inhibiting oxidative phosphorylation (OXPHOS) (50).

In conclusion, significant gaps remain in our understanding of the glycolysis mechanism in macrophages regulated by the PAM pathway under sepsis. Notably, the interaction between upstream PI3K-AKT signaling and downstream mTOR within the PAM pathway involves complex molecular networks, and their combined regulation of glycolysis is not absolute. Multiple factors can affect this regulation. For example, similar to the induction by the Toll-like receptor 4 (TLR4) agonist lipopolysaccharide, nucleotide-binding oligomerization domain 1 (NOD1) and nucleotide-binding oligomerization domain 2 (NOD2) agonists have been shown to trigger early glycolytic reprogramming in human monocyte-derived macrophages (MDMs), whereas this glycolytic reprogramming relies on Akt kinase but is independent of mTOR complex 1 (51).

### 2.3 NF-κB

NF-κB constitutes a family of transcription factors that play essential roles in multiple physiological and pathological processes. NF-κB is well known to be activated in response to diverse external stimuli and plays a critical role in inflammation and immune responses (52).

NF-κB plays a pivotal role in driving glycolysis in macrophages during sepsis by facilitating the expression of glycolysis enzymes via the transcription factor E2F1 (**Figure 1**) (53) and by enhancing glycolytic glucose flux via increasing glucose transporter type 6 (GLUT6) expression (54). Additionally, the inhibition of NF-κB reduces glycolysis and decreases the secretion of inflammatory factors, which may contribute to immunosuppression in sepsis. For example, high levels of NOD-like receptor family CARD domain-containing-3 (NLRP3) inhibit the binding of the promoter of nuclear NF-κB p65 and nuclear factor of activated T cells 5 (NFAT5) (**Figure 1**), thereby limiting the expression of glycolysis genes and pro-inflammatory cytokines in immunosuppressive macrophages (55).

Several studies have investigated upstream molecules that modulate NF-κB-mediated glycolysis in sepsis macrophages. B-cell lymphoma 3 (Bcl-3), an atypical member of the IκB protein family, can promote or inhibit the expression of NF-κB target genes according to the received cell type and stimulation (56) (**Figure 1**). Although it is an environment-dependent cell response regulator (57), recent studies found that the downregulation of Bcl-3 in LPS-stimulated macrophages enhances NF-κB pathway activation. So that it can regulate the expression of glycolysis-related genes (58).It is also observed in Th17 cells that Bcl-3 deficiency increased glycolysis (57). Moreover, intracellular ubiquitin-like modifications can also regulate NF-κB function. For example, neural precursor cell-expressed developmentally downregulated 8 (NEDD8, a ubiquitin-like protein)-mediated neddylation of Cullin1, a substrate of neddylation, promotes glycolysis and M1 polarization in macrophages through the NF-κB p65 pathway (**Figure 1**), thereby exacerbating the inflammatory response in sepsis (59). Interestingly, the same molecule can exert distinct effects depending on its cellular localization. Extracellular sequestosome 1 (SQSTM1/p62), an autophagy receptor, binds to the insulin receptor and activates the NF-κB-dependent metabolic pathway, leading to aerobic glycolysis and macrophage polarization. In contrast, intracellular SQSTM1 has no observed effect on glycolysis (60).

### 2.4 Mitochondrial functional damage

Mitochondria are the main energy-generating organelles in cells. Two core processes are involved in ATP production via glucose catabolism—glycolysis and the mitochondrial tricarboxylic acid cycle (TCA cycle, also called the Krebs cycle or citric acid cycle). The TCA cycle is tightly connected to glycolysis. Pyruvate is produced during glycolysis and converted into acetyl coenzyme A, which could enter the TCA cycle and drive OXPHOS (61). When immune activation and inflammation become excessive, mitochondrial function will be impaired (62, 63). This impairment disrupts the TCA cycle and shifts cellular energy metabolism toward aerobic glycolysis. Relevant studies support this conclusion that mitochondrial dysfunction and enhanced aerobic glycolysis happen at the same time in septic macrophages (64–66). In turn, restoring mitochondrial function usually weakens aerobic glycolysis (67). This implies that mitochondrial homeostasis may play a role in regulating aerobic glycolysis in inflammation. Part of this phenomenon stems from the accumulation of metabolic intermediates caused by TCA cycle disruption.

When mitochondrial function is impaired, the primary accumulated intermediate metabolites include citrate and succinate. Metabolites derived from citrate, such as oxaloacetic acid, indirectly induce the production of intracellular reactive oxygen species (ROS) and nitric oxide (NO). Elevated ROS leads to continuous oxidation of exposed methionine residues (M44) on glyceraldehyde 3-phosphate dehydrogenase (GAPDH), an important glycolytic enzyme, resulting in GAPDH aggregation and glycolysis promotion (**Figure 1**) (68). Meanwhile, upregulated NO promotes the S-nitrosylation and the activation of HIF-1α, thereby enhancing glycolysis (**Figure 1**) (69). In hypoxia and immune cell activation, the TCA cycle breaks down and cytosolic succinate accumulates (70). Emerging research indicates that inhibiting succinate dehydrogenase (SDH) will lead to intracellular succinate accumulation. Elevated levels of cytosolic succinate induce epigenetic alterations, mitochondrial ROS production (71) and PHDs inhibition (72, 73). It consequently stabilizes HIF-1α to upregulate aerobic glycolysis (**Figure 1**).

However, itaconic acid, another metabolite of citric acid, alkylates cysteine residue 22 on the glycolytic enzyme GAPDH (**Figure 1**) to inhibit its enzymatic activity and suppress aerobic glycolysis in activated macrophages (74). It can also work as an SDH inhibitor to suppress stabilization of hypoxia-inducible factor (HIF) to block glycolysis (75).

These findings suggest that mitochondrial function modulates both the activation and suppression of glycolysis and represents a potential therapeutic target for sepsis. Future studies should further investigate this direction.

### 2.5 Cytokines

Cytokines are the products of the body’s immune system and can stimulate macrophages. Mantovani and colleagues classified the stimuli into a continuum between two functionally polarized states based on their effects on selected macrophage markers, termed M1 (stimulated by interferon-γ (IFN-γ) combined with lipopolysaccharide (LPS) or tumor necrosis factor (TNF)) and M2 (stimulated by Interleukin-4 (IL-4), Interleukin-4 (IL-10), and glucocorticoids (GCs)) (76). The different types of macrophages activated by different cytokines exhibit two kinds of distinguishing metabolic and immune characteristics. Usually, the M1 type mainly shows rapid activation with early aerobic glycolysis and increased secretion of pro-inflammatory cytokines, such as Interleukin-1β (IL-1β), while the M2 type shows enhanced OXPHOS and attenuated glycolysis, and highly expresses chitinase 3-like 3 (Chi3l3) (also known as Ym1) and Fizz1 (also known as RELMa), participating in the inhibition of various inflammatory and allergic reactions as well as tissue repair (27, 77).

**Figure 2:**
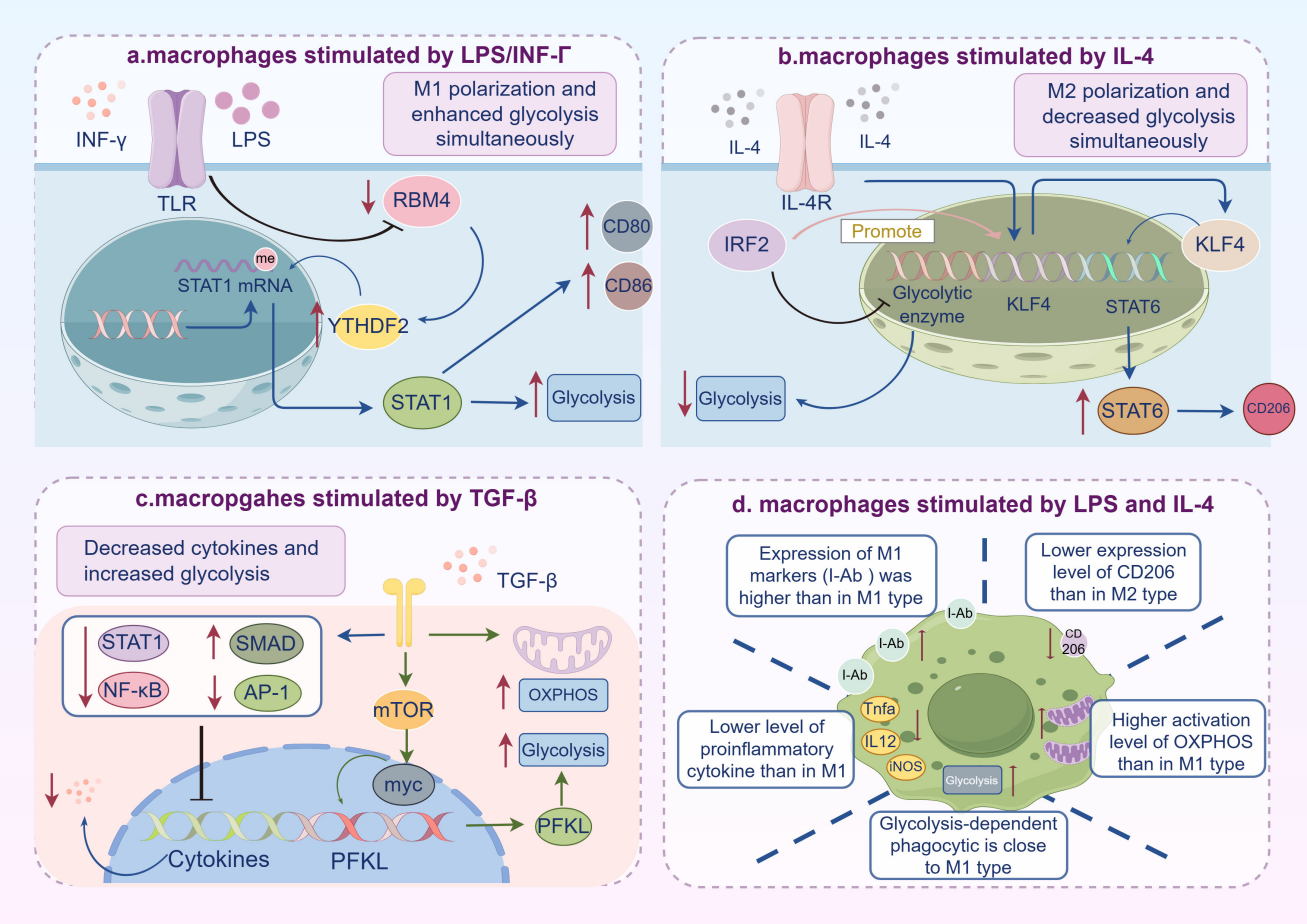
Distinct alterations in glycolytic activity and polarization states of macrophages induced by various cytokines stimuli. **(a)** Macrophages stimulated by LPS/IFN-γ tend to polarize toward M1 phenotype with enhanced glycolysis: IFN-γ downregulates RBM4 expression, thereby reducing YTHDF2-mediated inhibition and increasing m6A-modified STAT1 mRNA levels to promote glycolysis and M1 polarization simultaneously. **(b)** Macrophages stimulated by IL-4 tend to adopt an M2 phenotype with reduced glycolysis: IRF2 inhibits glycolysis by suppressing glycolytic gene expression and enhances STAT6 transcriptional activation and M2 polarization by promoting IL-4-induced KLF4 expression. **(c)** Macrophages stimulated by TGF-β tend to adopt an M2 phenotype with increased glycolysis and OXPHOS: TGF-β activates the transcriptional coactivator SMAD3 while suppressing pro-inflammatory transcription factors such as AP-1, NF-κB, and STAT1 to reduce cytokine secretion. Additionally, TGF-β enhances glycolysis via the mTORC1-MYC pathway. **(d)** Macrophages stimulated by both LPS and IL-4 exhibit a mixed M1/M2 phenotype: expression of M1 surface markers like I-Ab is higher than in M1 macrophages, but the expression of iNOS and several M1-associated genes like TNFα and Il12b are reduced. The M2 marker CD206 is expressed at lower levels in LPS/IL-4-induced macrophages compared to M2 macrophages. LPS/IL-4-induced macrophages exhibit high levels of glycolysis, with glycolysis-dependent phagocytic activity significantly enhanced. (by Figdraw).

However, it is too simplistic to make a direct correlation between a high level of aerobic glycolysis and an increased inflammatory response. On the one hand, their relationship is influenced by the effects of various cytokines. It has been demonstrated that the transforming growth factor-β (TGF-β) promotes glycolysis in macrophages while suppressing the production of inflammatory cytokines and impacts the survival of septic mice (**Figure 2c**) (78). Another study certified that macrophages stimulated by LPS and IL-4 exhibit significantly enhanced glycolysis-dependent phagocytic activity, resembling M1 macrophages. However, compared to M1 or M2 macrophages, LPS/IL-4-induced macrophages simultaneously exhibit elevated glycolytic and OXPHOS activities (**Figure 2d**) (77). On the other hand, the relationship also varies with cell type. Alveolar macrophages exhibit a markedly lower ability to respond to the typical type 2 cytokine IL-4 compared to lung tissue or peritoneal macrophages (79). Moreover, the origin of macrophages also plays a significant role. Airway macrophages (AM), the tissue-resident macrophages, exhibit greater sensitivity to IFN-γ stimulation compared to monocyte-derived macrophages and manifest as relying on glycolysis for cytokine secretion, whereas MDMs display lower sensitivity but a glycolysis-dependent upregulation of HLA-DR and CD40 (80).

The mechanisms by which cytokines link macrophage polarization to energy metabolism remain incompletely understood. Current studies indicate that cytokine stimulation activates polarization-related signaling pathways in macrophages, some of which intersect with glycolytic signaling pathways. IFN-γ promotes M1 macrophage polarization by activating activator of signal transducer and activator of transcription 1 (STAT1) (76), a transcription factor that facilitates IFN-γ-induced immune responses (81). Coincidentally, RNA-binding motif 4 (RBM4), a multifunctional RNA-binding protein, plays important roles on mRNA alternative splicing and translation control, has been shown to degrade m6A-modified STAT1 mRNA by interacting with YTH domain family protein 2 (YTHDF2), leading to the decrease of glycolysis. Notably, RBM4 expression is regulated by IFN-γ (82), suggesting that IFN-γ could connect macrophage polarization to aerobic glycolysis via the STAT1 pathway (**Figure 2a**). Similarly, interferon regulatory factor 2 (IRF2) inhibits glycolysis by suppressing glycolytic gene expression but promotes macrophage M2 polarization. It enhance STAT6-mediateded transcription, a key pathway for M2 polarization (76), which in turn facilitates IL-4-induced expression of Kruppel-like factor 4 (KLF4) (**Figure 2b**) (83). KLF4 is a transcription factor that regulates downstream gene expression and nuclear protein transcription through interactions with coactivators or immune complexes. Metallothionein 3 (MT3) is also another molecule potentially linking these processes. MT3 promotes the M2 macrophage phenotype, suppresses the pro-inflammatory M1 program, and inhibits hypoxia-inducible factor 1α (HIF-1α) activation (84), a key driver of aerobic glycolysis, as mentioned before. In macrophages stimulated by TGF-β, glycolysis increases, which is mediated by the mTORc-MYC-dependent pathway, while cytokine production decreases due to the activation of the transcriptional coactivator SMAD family member 3 (SMAD3) and suppression of the activity of the pro-inflammatory transcription factors sucn as activator protein 1(AP-1), NF-κB, and STAT1 (**Figure 2c**) (78).

## 3 Glycolytic enzymes or metabolites affecting inflammation

Since glycolysis was discovered to be closely associated with inflammation, researchers have continued to study it extensively. Here, we review the mechanisms by which glycolysis-related enzymes and metabolites in macrophages regulate inflammation in sepsis, as well as potential therapeutic targets for sepsis treatment.

### 3.1 Hexokinase 2

**Figure 3:**
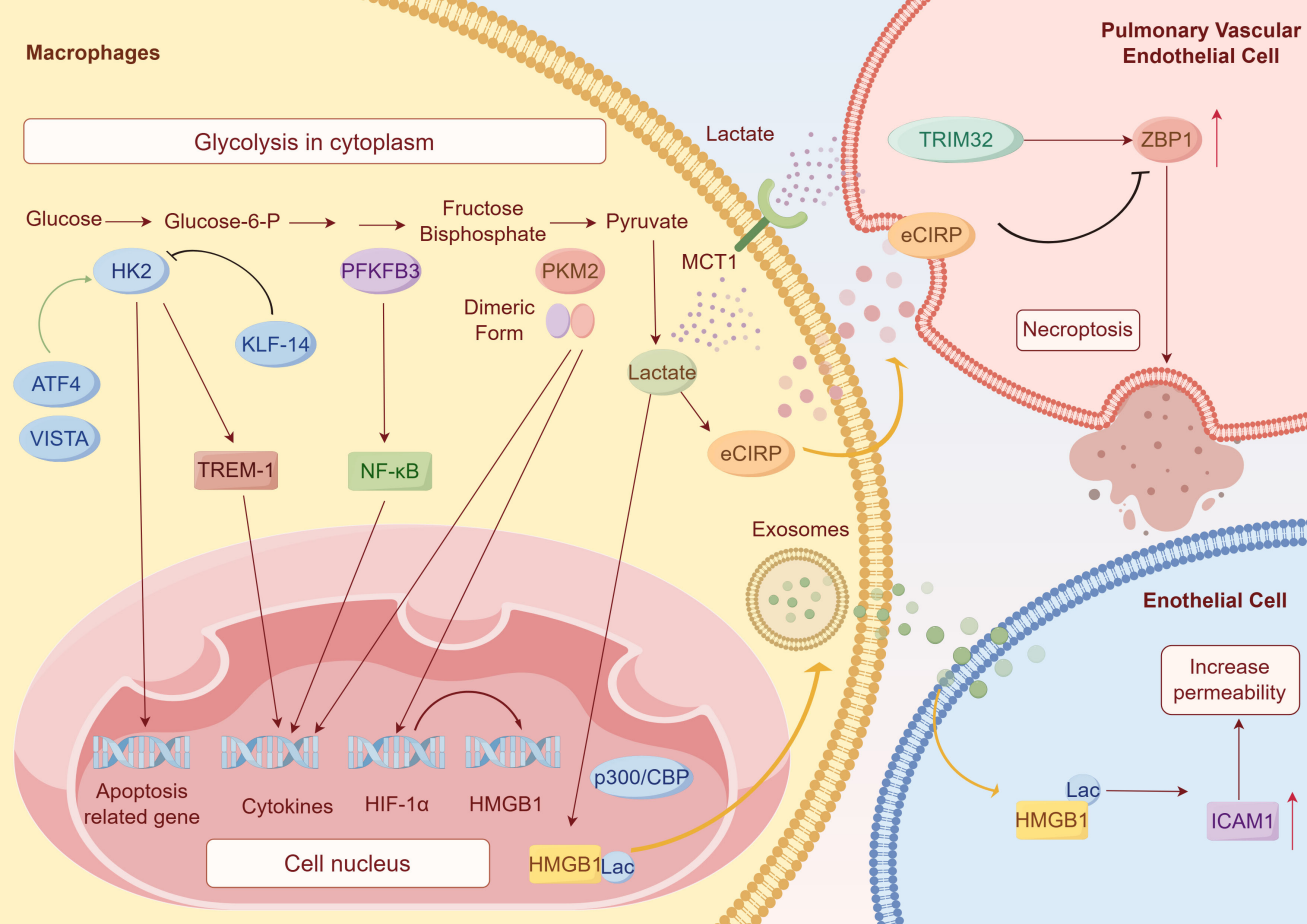
Glycolytic enzymes and lactate regulate inflammation. The flow chart in the figure represents the process of glycolysis of glucose to lactic acid: HK2 catalyzes the production of G-6-P from glucose, followed by the PFKFB1-catalyzed synthesis of fructose-2,6-bisphosphate, which then undergoes a series of changes that culminate in the production of lactate with the participation of enzymes including PKM2. HK2 regulates apoptosis-related genes, promoting NLRP3 inflammasome activation and cytokines secretion by inducing TREM-1. VISTA and ATF4 enhance HK2 expression, whereas KLF14 induced by IL-4 suppresses HK2 transcription. PFKFB3 promotes the LPS-induced pro-inflammatory gene expression by the NF-κB signaling pathway. PKM2 enhances HIF-1α expression by binding to its promoter to upregulate the expression of HMGB1 and IL-1β. Lactate mediates HMGB1 lactylation via acetyltransferase p300. Lactylated HMGB1 is secreted through exosomes, upregulating ICAM1 expression and increasing endothelial permeability. Meanwhile, lactate promotes the release of eCIRP from macrophages to PVECs to disrupt the interaction between ZBP1 and TRIM32, thus stabilizing ZBP1 and promoting necroptosis of PVEC. (by Figdraw).

Hexokinases (HKs), the first key rate-limiting enzymes in glucose metabolism, catalyze the conversion of glucose to glucose-6-phosphate (G-6-P), representing the first committed step of glucose metabolism (**Figure 3**) (85). Four major HK isoforms (HK1-4) are expressed in mammalian tissues, and several studies have demonstrated that HK2 is the primary isoform governing glycolytic metabolism in inflammatory macrophages. Its dysregulation is closely linked to abnormal immune function of macrophages in sepsis.

HK2 plays a pivotal role in controlling macrophage-driven inflammatory responses in sepsis via three main mechanisms: influencing inflammatory cytokine release, controlling NLRP3 inflammasome activation, and involved in the differentiation of monocytes into macrophages. Recent studies have shown that lidocaine reduces TNF-α and IL-6 secretion in LPS-stimulated macrophages, which is associated with reducing the expression of GLUT1 and HK2 (**Figure 3**) (85). Alatshan A’s team has verified that all-trans retinoic acid (ATRA) enhances HK2 expression and shifts the metabolism of LPS-activated macrophages toward glycolysis, resulting in NLRP3 inflammasome activation and enhanced IL-1β secretion (**Figure 3**) (45). Similarly, another study found that the glycolysis inhibitor 2-deoxyglucose (2-DG) suppresses TREM-1-induced NLRP3 inflammasome activation (50), as 2-DG is a hexokinase inhibitor (86–88). Monocyte chemoattractant protein-1 (MCP-1), also known as CCL2, is a member of CC chemokines. It promotes migration and infiltration of inflammatory cells like monocytes/macrophages and other cytokines at the site of inflammation (89). This function is markedly attenuated by 2-DG (47), which is also potentially regulated by Akt-dependent metabolic reprogramming.

In addition to the well-known metabolic regulators HIF-1α (87) and mTOR (50), several studies have identified additional upstream regulators of HK2, offering potential therapeutic targets for modulating glycolysis in sepsis treatment. V-domain immunoglobulin suppressor of T cell activation (VISTA) regulates HK2 expression (**Figure 3**) through K63-linked ubiquitination mediated by the E3 ubiquitin ligase TRIM28, thereby modulating the microglial activation in sepsis-related encephalopathy. Treatment with a VISTA antibody significantly reduces microglial activation in septic mice and prevents cognitive impairment (88). KLF4, suppresses HK2 transcription by binding to its promoter (**Figure 3**) thereby reducing glycolysis and inflammatory cytokine secretion in macrophages (90). Another transcription factor, activating transcription factor 4 (ATF4), enhances HK2 expression by binding to its promoter and interacting with HIF-1α (**Figure 3**). Notably, ATF4 overexpression improved immune tolerance, which may be relevant to the reduced expression of HK2 mRNA and protein as well as decreased lactate release (87). It shows that ATF4, a crucial upstream regulator of HK2, can influence HK2-driven glycolysis during both the hyperinflammatory and immunosuppressive stages of the disease.

### 3.2 Fructose-2,6-bisphosphatase

Fructose-2,6-bisphosphatase (PFKFB) synthesizes fructose-2,6-bisphosphate. During glycolysis, PFKFB promotes fructose-2,6-bisphosphate synthesis, which has a positive feedback effect to enhance the catalytic activity of PFK, thereby accelerating glycolysis. Studies have demonstrated that the activity of PFKFB3 could be increased through phosphorylating PFKFB3 protein (91) and its expression could be elevated by zinc fingers and homeoboxes 2 (Zhx2) binding to the promoter (92), both of which finally promote glycolysis and pro-inflammatory cytokines, including TNF-α, IL-6, and IL-1β.

PFKFB3 promotes inflammation through multiple mechanisms. It could facilitate the expression of inflammatory factors. Studies have shown that PFKFB3 ablation or inhibition with AZ26, a Pfkfb3 inhibitor, suppresses LPS-induced pro-inflammatory gene expression by inhibiting the NF-κB signaling pathway (**Figure 3**) (93). Additionally, PFKFB3 could promote macrophage infiltration into the lungs in sepsis-induced ALI (94). In addition to enhancing macrophage infiltration, PFKFB3 reduces the expression of adhesion molecules intercellular adhesion molecule 1 (ICAM-1) and vascular cell adhesion molecule 1 (VCAM-1) in human pulmonary artery endothelial cells, thereby limiting macrophage infiltration (95). These findings suggest that PFKFB3 plays an important role in the regulation of macrophage infiltration into the lung from the capillaries during sepsis-induced ALI.

miR-29b-3p and miR-106a-5p are two kinds of small noncoding RNAs that can regulate gene expression posttranscriptionally. They have been confirmed to negatively regulate the expression of PFKFB3 in macrophages (96, 97). thereby reducing the glycolytic level and inflammatory response of macrophages and exerting a certain protective effect against organ dysfunction caused by LPS. This also provides us with a new approach for treating sepsis by targeting PFKFB3.

### 3.3 Pyruvate Kinase

Pyruvate kinase, a rate-limiting enzyme in glycolysis, catalyzes the conversion of phosphoenolpyruvate (PEP) to pyruvate (**Figure 3**). This reaction is catalyzed by the PKM1 isoform in differentiated tissues, whereas the PKM2 isoform mediates this process in tumors and inflammatory conditions (98), which exists in dimeric or tetrameric forms. Macrophages preferentially increase the dimeric form of PKM2, the form that promotes the Warburg effect, with LPS stimulation. PKM2 also enhances the expression of IL-1β and HIF-1α by binding to their promoters (**Figure 3**), thereby modulating immune responses (99). Studies have also demonstrated that PKM2 could upregulate high mobility group box 1 (HMGB1) by enhancing HIF-1α expression (**Figure 3**) (100). The circulating HMGB1 is a late cytokine, and distinguished from other early proinflammatory cytokines (101), which assists LPS reach caspase-11 to generate severe inflammation (102).

LPS-induced tyrosine phosphorylation together with increased PKM2 expression may promote PKM2 dimerization. This inhibits the formation of PKM2 tetramers and stabilizes its dimeric structure to facilitate the Warburg effect (99). This phenomenon was first identified in tumor cells. Recent sepsis-related studies have shown that inhibiting PKM2 phosphorylation in LPS-stimulated macrophages can lower the protein expression levels of HIF-1α and LDHA (103). This aligns with cancer research findings, where blocking phosphorylation helps PKM2 form tetramers. Even though PKM2 phosphorylation is a potential therapeutic target for sepsis, more experimental verification is still needed.

The tetrameric form of PKM2 has been consistently identified as a key driver of M2 macrophage polarization both *in vivo* and vitro (99, 104, 105). Mechanistically, tetrameric PKM2 enhances mitochondrial biogenesis through PGC-1α by suppressing the PI3K/Akt signaling pathway. And it fosters endotoxin tolerance by reducing the release of proinflammatory cytokines TNF-α and IL-6 in laboratory settings and *in vivo (*104). Additional evidence from a sepsis-induced liver injury model supports this finding. The natural compound Forsythoside E (FE) binds to the PKM2 K311 residue, promoting PKM2 tetramerization. This tetrameric PKM2 then encourages M2 polarization by inhibiting STAT3 phosphorylation and NLRP3 inflammasome activation, while also restoring mitochondrial membrane potential and morphology (105). This is also proved in CLP mice. The biological impact of PKM2 tetramer-driven endotoxin tolerance varies with the context. In hosts with a healthy immune system, this tolerance helps reduce excessive inflammation and organ damage during the hyperinflammatory stage of sepsis (104). Conversely, extended endotoxin tolerance in immunocompromised individuals, such as the elderly or post-septic patients, can weaken anti-microbial defenses, leading to a higher risk of secondary infections and increased sepsis-related mortality (99).

This duality underscores the importance of precise temporal targeting of PKM2 tetramerization: to activate it during the hyperinflammatory phase while avoiding prolonged activation that could potentially intensify immunosuppression.

### 3.4 The glycolytic metabolite, lactate

Lactic acid is a byproduct of glycolysis, which will build up when in sepsis under inflammatory conditions as a result of the Warburg effect. It also has a part to play in regulating immune responses. Histone lysine lactylation, which originates from lactic acid, is a newly identified epigenetic modification. Hypoxia and bacterial infection can induce glycolysis, leading to lactic acid production, and this lactic acid acts as a precursor for histone lactylation (106). This finding underlines that lactic acid is not just a metabolic intermediate—it also directly regulates gene expression via protein modification.

Severe hyperlactatemia is often linked to septic shock (107). Clinical research has found that histone H3 lysine 18 lactylation (H3K18la) levels show a positive correlation with disease severity in septic patients (108). This clinical correlational observation implies that H3K18la may mediate the expression of inflammatory factors in septic macrophages, which may further regulate the immune function of these cells. For example, it is found that intracellular lactate enhanced the activation of the NF-κB signaling pathway through the p300/CBP-mediated lactylation of H3K18 (109). H3K18la can also promote the expression of interferon regulatory factor 7 (Irf7), a key regulator of type I interferon responses. *In vivo* experiment, inhibiting lactylation of H3K18 can reduce multi-organ inflammation and enhance survival (110).

With further research, lactylation is not only observed in histones but also in non-histone proteins, influencing the pathological progression of diseases. Elevated vascular permeability is a common feature of sepsis (111). Recent studies indicate that macrophages take part in this pathophysiological process by lactylation with glycolysis-derived lactate. Lactate in macrophages is transported into the nucleus to promote the lactylation of HMGB1 with the help of acetyltransferase p300. Lactylated or acetylated HMGB1 is then secreted by macrophages via exosomes, enhancing endothelial permeability (**Figure 3**) (112). Furthermore, a recent study found that lactate takes part in regulating macrophage pyroptosis in sepsis. It identified NLRP3 as a target protein modified by lactate to facilitate inflammasome activation in macrophages, which is lactylated by AARS2. *In vivo*, inhibition of lactate production alleviates inflammatory responses in polymicrobial sepsis (113).

Lactate and lactylation also contribute to the pathological processes of sepsis complications. Lungs are among the most susceptible organs in sepsis. Patients with sepsis-induced ALI have higher mortality and poorer prognosis (114). Recent studies show that hyperlactatemia drives macrophages to release extracellular cold-inducible RNA-binding protein (eCIRP). Pulmonary vascular endothelial cells (PVECs) internalize eCIRP through Toll-like receptor 4 (TLR4)-mediated endocytosis. This process disrupts the interaction between ZBP1 (Z-DNA binding protein 1) and tripartite motif-containing 32 (TRIM32), stabilizing ZBP1 and further promoting PVEC necroptosis (**Figure 3**) (115). AKI is another common sepsis complication. Experimental studies suggest that glycolysis-derived lactate in human kidney 2 cells (HK-2 cells) regulates the molecular mechanisms behind AKI progression (116). Still, the role of macrophages here remains unclear, and research of lactylation changes in sepsis is still in its early phases. Recent studies have shown that lactated HMGB1 inside macrophages can trigger neutrophil extracellular trap formation through the cGAS/STING pathway, potentially contributing to the mechanisms by which macrophages partake in SAKI development (117). Lactation modification has emerged as a focal point of mechanism research over the past few years, with significant gaps that remain to be explored.

## 4 Research gaps and therapeutic potential

Numerous studies have clarified the crosstalk between inflammation and glycolysis in septic macrophages, but many questions still remain unanswered. This complexity stems from the multi-level interactions between inflammation and glycolysis—these involve multiple signaling pathways and an intricate intracellular molecular network, including inflammatory factors, tricarboxylic acid (TCA) cycle metabolites, and glycolytic metabolites. As we noted earlier, current research indicates that the roles of these molecules differ depending on cell type and the complex regulatory network within cells. The inflammatory response also exhibits temporal heterogeneity. The functions of these molecules and pathways undergo dynamic changes at different stages of sepsis (118). In addition, although most studies have focused on the interplay between glycolysis and M1/M2 macrophage polarization during inflammation, macrophages usually exist as mixed subtypes under disease conditions and are regulated by multiple factors. This makes the *in vivo* crosstalk between glycolysis and macrophage polarization highly complex. For example, a new study identified a subpopulation of monocyte-derived cardiac macrophages, termed iNOS(+) Arg1(+) macrophages, which simultaneously express pro-inflammatory and pro-reparative genes in exercised male mice. Exercise enhances glycolysis in monocytes, increasing lactate production and driving histone lactylation at H3K18. H3K18la accelerates the transition of cardiac macrophages to a pro-reparative state, restoring immune homeostasis and preserving cardiac function (21). In this study, moderate promotion of glycolysis facilitates disease recovery in turn, which may be related to different macrophage subtypes.

In addition to the fact that more research is needed to explore the mechanism, drug therapy targeting glycolysis faces the same dilemma. Although recent studies have demonstrated that several drugs improve survival rates in septic mice by inhibiting glycolytic enzymes, including LDHA (119), HK2, and PFKFB3 (120), research in this area remains in its early stages, except for some detailed studies on PKM2. Celastrol (CEL), a natural anti-inflammatory compound, inhibits PKM2 by binding to Cys424 to suppress aerobic glycolysis and reduces IL-1β secretion by binding to Cys106 of HMGB1 (121). Meanwhile, it is indicated that deoxyelephantopin downregulates PKM2 mRNA and protein expression in a dose-dependent manner and inhibits PKM2 nuclear localization, thereby attenuating glycolysis and reducing IL-1β and HMGB1 levels (122). In the above studies, septic mice treated with drugs received protection from lethal endotoxemia and improved survival rates. Additionally, some studies have shifted focus from glycolytic enzyme inhibitors to the TCA cycle. Chicoric acid (CA) was identified as the most potent inhibitor of SDH activity in macrophages among 179 phenolic compounds. When ATP turnover rate decreases caused by factors such as inflammation-induced mitochondrial dysfunction, the demand for NAD^+^ (Nicotinamide adenine dinucleotide) to support oxidation reactions in cells is greater than the ATP turnover rate, and cells may engage in aerobic glycolysis (123). Pre-treatment with CA reduces glycolysis by inhibiting SDH to meet NAD^+^ regenerative demand and increase the NAD^+^/NADH ratio in activated macrophages (124).

Although basic research has identified multiple potential therapeutic targets, most of these mechanistic insights are derived from single-factor stimulated (e.g., LPS) isolated macrophage models, which cannot fully mimic the polymicrobial infection, dynamic tissue microenvironments and temporal immune heterogeneity of *in vivo* sepsis. Thus, the translation of these glycolytic targeting strategies into clinical practice is hindered by the lack of *in vivo* validation under physiological complex conditions.

In summary, the crosstalk between inflammation in sepsis and glycolysis in macrophages is super complicated, with multiple molecules participating in it, bringing many difficulties to be solved. In the future, a deeper understanding of the regulatory mechanisms in sepsis macrophages is expected to provide more effective treatment methods for sepsis patients and improve their prognosis.

## Glossary

2-DG: 2-deoxyglucose
AKI: Acute kidney injury
AKT: Protein kinase B
ALI: Acute lung injury
AM: Airway macrophages
AP-1: Activator protein 1
ATF4: Activating transcription factor 4
Bcl-3: B-cell lymphoma 3
bHLH: Basic helix-loop-helix
BMDMs: Bone marrow-derived macrophages
CA: Chicoric acid
CD206: Cluster of differentiation 206
CEL: Celastrol
Chi3l3: Chitinase 3-like 3
E2F1: E2F transcription factor 1
eCIRP: Cold-inducible RNA-binding protein
G-6-P: Glucose-6-phosphate
GAPDH: Glyceraldehyde 3-phosphate dehydrogenase
GCs: Glucocorticoids
GLUT6: Glucose transporter type 6
HIF-1α: Hypoxia-inducible factor-1α
HK2: Hexokinases 2
HLA-DR: Human leukocyte antigen-DR
HMGB1: High mobility group box 1
HREs: Hypoxia response elements
ICAM-1: Intercellular adhesion molecule 1
IFN-γ: Interferon-γ
IL-10: Interleukin-10
IL-1β: Interleukin-1β
IL-4: Interleukin-4
iNOS: Inducible nitric oxide synthase
IRF2: Interferon regulatory factor 2
KLF14: Kruppel-like factor 14
LDHA: Lactate dehydrogenase A
LPS: Lipopolysaccharide
MCP-1: Monocyte chemoattractant protein-1
MDMs: Monocyte-derived macrophages
METTL3: Methyltransferase-like 3
MSC: Mesenchymal Stem Cell
MT3: Metallothionein 3
mTOR: Mammalian target of rapamycin
NAD+: Nicotinamide adenine dinucleotide
NADH: Nicotinamide adenine dinucleotide hydrogen
NEDD8: Neural precursor cell-expressed developmentally downregulated 8
NFAT5: Nuclear factor of activated T cells 5
NF-κB: Nuclear factor kappa-B
NLRP3: NOD-like receptor family CARD domain-containing-3
NMDA: N-methyl-D-aspartate
NMDAR: N-methyl-D-aspartate receptors
NO: Nitric oxide
NOD1: Nucleotide-binding oligomerization domain 1
NOD2: Nucleotide-binding oligomerization domain 2
OXPHOS: Oxidative phosphorylation
PAM: PI3K-AKT-mTOR
PAS: PER-ARNT-SIM
PEP: Phosphoenolpyruvate
PFK: Phosphofructokinase
PFKFB: Fructose-2,6-bisphosphatase
PHDs: Prolyl hydroxylases
PI3K: Phosphatidylinositol 3-kinases
PKM2: Pyruvate kinase M2
PVECs: Pulmonary vascular endothelial cells
RBM4: RNA-binding motif 4
ROS: Reactive oxygen species
SAE: Sepsis-associated encephalopathy
SDH: Succinate dehydrogenase
SETD2: SET domain containing 2
SMAD3: SMAD family member 3
SQSTM1/p62: Sequestosome 1
STAT1: Signal transducer and activator of transcription 1
STAT6: Signal transducer and activator of transcription 6
TCA: Tricarboxylic acid cycle
TGF-β: Transforming growth factor-β
TLR4: Toll-like receptor 4
TNF: Tumor necrosis factor
TREM-1: Triggering receptor expressed on myeloid cells-1
TRIM28: Tripartite motif containing 28
TRIM32: Tripartite motif-containing 32
VCAM-1: Vascular cell adhesion molecule 1
VISTA: V-domain immunoglobulin suppressor of T cell activation
YTHDF2: YTH domain family protein 2
ZBP1: Z-DNA binding protein 1
Zhx2: Zinc fingers and homeoboxes 2
